# Nationwide citizen access to their health data: analysing and comparing experiences in Denmark, Estonia and Australia

**DOI:** 10.1186/s12913-017-2482-y

**Published:** 2017-08-07

**Authors:** Christian Nøhr, Liisa Parv, Pille Kink, Elizabeth Cummings, Helen Almond, Jens Rahbek Nørgaard, Paul Turner

**Affiliations:** 1 Danish Centre for Health Informatics, Department of Development and Planning, Aalborg University, Vestre Havnepromenade 5, 9000 Aalborg, Denmark; 2 E-Health Laboratory, Tallinn University of Technology, Tallinn, Estonia; 3 East Tallinn Central Hospital, Tallinn, Estonia; 4 School of Health Sciences, University of Tasmania, Hobart, Australia; 5 eHealth Services Research Group, School of Engineering & ICT, University of Tasmania, Hobart, Australia; 6 MedCom, The Danish Health Data Network, Odense, Denmark

**Keywords:** Electronic health record, Medical informatics application, Patient centred care, Health services

## Abstract

**Background:**

Most countries face an ageing population, increasing chronic diseased, and constrictions on budget for providing health services. Involving patients in their own care by allowing them access to their patient data is a trend seen in many places.

**Methods:**

Data on the type and level of access citizens have to their own health data in three countries was gathered from public sources.

**Results:**

Data from each individual country is presented and the experiences of Denmark, Estonia and Australia are examined whilst similarities and differences explored. The discussion adopts a citizen-centred perspective to consider how the different e-portal systems support, protect and structure citizen interactions with their own health data in three key areas: Security, privacy and data protection; User support; and Citizen adoption and use.

**Conclusions:**

The paper highlights the impact of opt-in/opt-out approaches on citizen access and the lack of a structured approach to addressing differences in citizen health and e-health literacy. This research also confirms while current data provides detail on the availability and use of personal health data by citizens, questions still remain over the ultimate impact on patient outcomes of these initiatives. It is anticipated the insights generated from the three countries experiences, supporting citizen access to their health data will be useful to improve these initiatives and guide other countries aspiring to support similar initiatives.

## Background

Most countries face an ageing population, increasing prevalence of chronic diseases and experience an ever-tightening budget for providing health services. Involving patients in their own care by allowing them access to their patient data is a trend seen in many places. A number of commercial solutions allow individual users to gather, store, use, and share health information are offered for example by Microsoft through its HealthVault – started in 2007. Google launched a similar product – “Google Health” - in 2008, but discontinued the service in January 2012 because it was not having the broad impact that they hoped it would [1].

Some Health Maintenance Organizations (HMO’s) have developed their own personal health record (PHR) systems for their members to use. For instance the Veterans Administration’s (VA) “My Health***e***Vet” is the VA’s online personal health record, and Kaiser Permanente offers a system called “My Health manager”. Other HMO’s apply extensions to commercial Electronic Health Record products (e.g. Cerner Health or Epic’s MyChart) to allow patients access their own health data.

It is however, noticeable that only a few countries governments have offered citizens access to their own health data on a national level. Through a search of official national government, or national health organizations’ web sites, a number of countries offering their citizens access to their personal health related data were identified. Australia, Canada, Denmark, Estonia, Finland, France, Iceland, New Zealand, Norway, Scotland, Singapore, and Sweden, are in a phase where they plan (or already do) provide citizen access to their health data. However, at the time of writing this manuscript only three countries have had systems established for a period of more than two years and consequently have data on the usage of the facilities.

Denmark, Estonia and Australia are three countries that have been identified where all citizens currently have access to their personal health data via a e-portal solution.

Many other countries have tried and failed, and are in the process of developing systems allowing citizens to access their own patient data. For example, in Britain an internet accessible personal electronic health record was first introduced in the English National Health Service (NHS) in 2007. The project called “HealthSpace” was inspired by the Kaiser model. From the launch in 2007 to October 2010, 172,950 people opened a basic HealthSpace account [2]. HealthSpace closed on 14th December 2012 because the service was not as popular as the NHS had anticipated [3]. More recently the UK has launched a strategy called “the power of information” where one key commitment is citizens will be able to view their GP record online by 2015. While France has enabled patient access to medical records via the Dossier Medical Personnel providing formal consent procedures are completed [4].

This paper presents data on the type and level of access citizens have to their own health data in three countries leading implementation in citizen access to their own health data. The experiences of Denmark, Estonia and Australia are examined and compared. It is anticipated that the insights generated from these three countries experiences will be useful to improve existing solutions and guide other countries aspiring to support similar initiatives.

## Methods

**Fig. 1:**
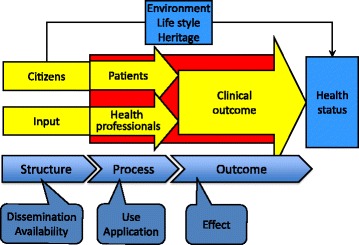
Conceptual framework of structure, process and outcome of a health care system

Describing health information systems (HIS) on national levels can be done in various ways. Donabedian found inspiration in systems theory and developed a structure-process-outcome framework to describe a generic health care system [5]. Figure 1 shows the health care system has structural components, buildings, equipment, IT-systems and staff.

The citizens become patients when they are admitted to professional health care. When these two entities are interacting in the health care process they produce a clinical outcome that eventually impacts the general health status. Indicators for national HIS can be described in terms of structural measures as well as process measures and effect measures. The ideal would be to measure effects and directly take readings of the results of the efforts and investments. However, effects are consequences of a sequence of events or actions and given the complexity of health care it can be difficult to determine what effects are caused by the use of a specific HIS.

It is more common to measure dissemination and availability by counting e.g. how many individuals have access to a specific HIS. But it becomes insignificant to quantify structural measures such, as access, when the implementation of a specific system approaches saturation. This is particularly true for national PHR systems as the very idea is to give everybody access to their health data. It is more interesting to quantify the process measures of the citizens who actually use the system in terms of e.g. how often users log on, and what do they look for?

In approaching this paper, the structural components of the systems providing citizen access are described in terms of the aim of the PHR system, its architecture, and security as well as user support and user characteristics. It is not possible at this stage to provide data on what specific information the citizens look up. Obviously, there are a lot of differences between the three systems, however the comparison in this study is focusing on the functionalities that are present in all three countries.

The specific data in the study has been acquired through official databases which is publicly available. The exact source is referenced where data is used.

## Results

### Description of structure characteristics

#### History, aim and functionalities of PHR

##### Denmark

**Fig. 2:**
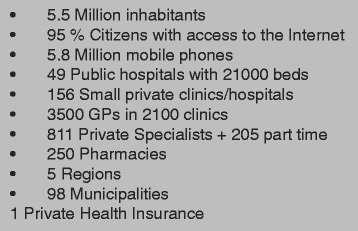
Basic information about Denmark [16]

The basic information about the Danish health care system can be seen in Fig. 2. The Danish National eHealth Portal is called “sundhed.dk” was launched in 2003 and was based on IBM WebSphere technology. In 2009 the eHealth portal was upgraded and re-launched on a new technical platform. It provides access to information in existing databases for citizens as well as health care professionals. In addition, the portal also gives free access to directories of health institutions in Denmark and a number of health related encyclopedia.

The official aims of the eHealth portal are [6]:

- Bring together relevant information from all parts of the health service
- Offer a shared platform of communication
- Empower patients by offering maximum insight and transparency in the health care sector
- Offer health care providers easy access to clinical information about their patients’ medical history.

All Danish citizens have access to sundhed.dk, enabling patients to get an overview of what health care data that exist in a number of specific databases.

Upon identification, the citizens can find accurate and updated health care information, e.g.:

- Online Electronic Health Record from hospitals – (eRecord)
- Cross-sectorial personal electronic medicine profile – (Shared Medication Record)
- Overview of personal medical history since 1977 (list of contacts with hospitals)
- Overview of contact with general practitioners (GP) since 2003

In addition the eHealth portal offers a number of services where the citizens can:

- Book appointments with their general practitioner
- Renew prescription drugs
- Monitor own drug compliance
- Survey shortest waiting lists for operations and quality ratings of hospitals
- Register as organ donor
- Get access to local disease management systems in out-patient Clinics.

In this study, we focus on E-Record and the Shared Medication Record.

##### Estonia

**Fig. 3:**
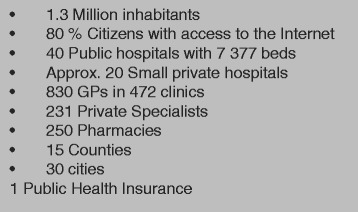
Basic information about Estonia

The basic information about the Estonian health care system can be seen in Fig. 3. The Estonian Patient Portal is part of the wider national e-Health framework in Estonia. In 2005, the Estonian Ministry of Social Affairs developed a strategy to create a more citizen-centric health care system through shared data across different levels of health care. The e-services aimed to improve quality by enabling better access and use of relevant health data as well as enhance health reporting and cost calculations [7]. The Estonian E-health Foundation is the central agent in charge of standardization and the development of digital medical documents. Relevant legal foundations of the system were set in 2007 with the regulations for data protection in electronic transmission.

The Patient Portal (PP) was created to enable citizens’ access to their data collected through the different e-Health services. The PP was established to enable patients to view the data gathered through the other four e-health services [8].

The Estonian Patient Portal (PP) enables people to view and modify personal information gathered from different public databases. The PP also includes information about one’s general practitioner and health insurance. It is possible to supplement and change existing information, however it does not change the information anywhere else but the Patient Portal.

The PP also enables to communicate permissions and requests to the government and health care providers, for example to allow to accept blood transfusions or becoming an organ donor. In addition, people assign others to become their trustees, allowing them to view personal medical information or purchase prescribed medicine on someone else’s behalf. All information about prescribed and bought medicine is available through the PP.

One of the central functionalities of the PP is the in- and outpatients care summaries that health care providers are obliged to send to the central platform. The deadlines for sending the information are five workdays for inpatient and one workday for outpatient care summaries. The health care providers send a summary for every outpatient case, not necessarily for every visit to the physician. Outpatient care summaries also include services provided in day surgery centres.

The PP also includes information about immunizations performed at the GP’s office and school. The birth summary is the first document inserted in the PP for a new-born. On top of outpatient summaries, children and young adults aged 0–19 have additional information in the PP concerning mandatory regular check-ups performed at the GP and in school.

The laboratory diagnostic test results are sometimes presented within the care summaries but a specific data standard is in place only since mid 2014. This means that as of mid 2015, all laboratory test results are included in the PP. Diagnostic images are stored in a separate database and accessible only for physicians. However, the image results with radiologist’s descriptions are available in the PP.

The latest functionalities added to the PP include a self-reported Health Declaration that each citizen should fill out. This includes information about one’s health status and health related behaviour. The document is compulsory to fill out for renewing one’s driving licence as additional information for the physician during the mandatory check-up. Moreover, as of 2015 national screening programs for breast and cervical cancer use the central health information data to develop their yearly sample population and send invitations for screening.

The future developments of the PP include services enabling patients to find the first available time for a primary or secondary care provider across the country. The patient will be able to book and cancel appointments through the PP. In the future, the PP will also include critical care summaries concerning contacts with the ambulance care team. The patient can also order reminders for appointments and send updated contact details to all health care providers from the PP.

##### Australia

**Fig. 4:**
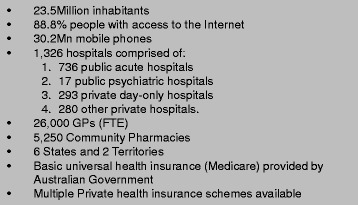
Basic information about Australia

The basic information about the Australian health care system can be seen in Fig. 4. The Australian electronic health record, formally named the Personally Controlled Electronic Health Record (PCEHR), renamed My Health Record (MyHR) 2015, was launched in 2012. The MyHR system and associated infrastructure was developed using a combination of ‘bottom up’ lead implementation projects and ‘top down’ national initiatives [9]. The architecture relies on a business-to-business gateway that provides the link between disparate systems and ensures interoperability [10]. MyHR integrates web based personal health records with a clinical electronic health record system and allows shared access to summary data for both consumers and healthcare providers based on shared responsibilities [11].

MyHR aims to ensure that the citizen is located at the centre of their healthcare by enabling them to access health information when and where it is needed. It is “an important systemic opportunity to enable person-centered care, support informed consumer decision making, improve quality and safety of care, reduce waste and inefficiency, and improve continuity and health outcomes for patients” [12].

Citizens have access to summary information about their medical history, medications, test results and allergies, which provide critical information for informed decision making about their healthcare. Records are available nation-wide and citizens are able to give permission for health professionals anywhere in the country to access their relevant history.

All Australian citizens, healthcare providers, and healthcare organisations are able to register to gain access to MyHR, but all parties must be registered for the benefits to be realized.

MyHR provides citizens with the opportunity to access summary health care data and share information with their health care providers. It is intended that citizens will have access to read, in full, everything that is added to their eHealth record by their health care providers. However, some functionality, such as access to diagnostic imaging, is still under development.

Citizens have their own section in the eHealth record where they can record personal information and notes for their own use. Some personal information, which typically includes information about allergies, adverse reactions, and current medications, are accessible by healthcare providers. However, personal notes are intended for patients’ private use and are not accessible by healthcare providers except if they are shared during a consultation through the personal portal. This information can contain, but is not limited to, information for monitoring health progress, non-prescription medications and other information relating to the individual’s health or healthcare.

Information held in Government registers including Medicare, Pharmaceutical Benefit Services, Organ Donor Status and the Australian Childhood Immunization Register can be linked, with MyHR and viewed by citizens and their health care providers.

Medication prescribing and dispensing information is available to individuals within their MyHR, it requires the prescribing clinician and the dispensing pharmacy to be registered and using MyHR. The medication information facility provides details of the medications prescribed, including the date, brand and generic names, the dose, and specific directions for use.

Parents of children under the age of 14 years can register and view their child’s MyHR. The child record is similar to the adult record but includes additional information regarding a child health development section where parents can add information pertinent to their child’s health, growth and development. There is a link to the immunization information.

There is also provision for “non- professional” carers to register, view and add to their family member or clients details.

#### Summary comparison of the patient portal functionalities

Based on analysis of portal functionalities Table 1 shows an overview of the functionalities in each of the patient portals in the three countries. For each functionality, the nature and type of communication supported is indicated across three levels. A summary of procedures for log-in and security is shown in Table 2.

**Table 1:** Summary of the functionalities in patient portals in Australia, Denmark and Estonia

Functionality available	Denmark	Estonia	Australia
Permissions for access	Opt-out	Opt-out	Opt-in
Non-medical information	Read and modify (Level 1)	Read and modify (Level 1)	Read and modify (Level 1)
Permissions and requests^a^	Read and modify (Level 1)	Read and modify (Level 1)	Read and modify (Level 1)
Immunization	Read and modify (Level 1)	Read (Level 2)	Read (Level 2)
Prescriptions	Read and renew (Level 1)	Read (Level 2)	Read (Level 2)
Outpatient Care summaries	Read (Level 2)	Read (Level 2)	Read (Level 2)
Referral letters (if applicable)	N/A	Read (Level 2)	Can be uploaded (Level 2)
Inpatient Care summaries	Read (Level 2)	Read (Level 2)	Read (Level 2)
Diagnostic laboratory tests	Read (Level 2)	Under development (Level 3)	Read (Level 3)
Diagnostic images	Not available to citizens (Level 3)	Read (Level 3)	Read (Level 3)
Appointments to primary care, secondary care physicians.	Read and book (Level 1)	Under development (Level 3)	
Log data on access	Read (Level 2)	Read (Level 2)	Read (Level 2)

^a^Read and modify one’s official representative(s). He or she has the right to read, modify data and/or fill in prescriptions depending on the extent of the rights given. Read and alter one’s volition or declination to donate organs, receive blood transfusion and donate one’s body to medical research. A Person has the right to take back the volition at any time, the volition in compulsory for physicians to abide by

**Table 2:** Summary of procedures for log-in and security

Log in and security issues	Denmark	Estonia	Australia
What is the log-in procedure	Two factor authentication	2 factor PKI combined with an electronic identity	MYGOV generated Number; Personally Generated Password; Security Name or Number (via Mobile Phone)
Who has access?	The citizens can access own data. Health professional can access data of patients they treat	The citizens can access own data. Health professional can access data of patients they treat	The citizens can access own data. Health professional can access data of patients they treat
How is security controlled?	Letter send in case of suspected abuse. Citizens control own log. Bi-annual audits of log files	Citizens are expected to report suspicious behaviour to the E-Health Foundation	Citizens are expected to report suspicious behaviour to the E-Health Foundation
User support	Only technical and navigation questions.	Only technical and navigation questions.	Only technical and navigation questions.

Level 1 - One-way communication - citizens are able to read information created by the health authorities or individual health care providers.

Level 2 – Two-way communication - citizens are able to modify existing information, receive reminders, book appointments, or renew prescription.

Level 3 – Communication currently under development - some system functionalities are under development and/or detailed information on the nature of how they will be used to support citizen access is not yet available.

#### Summary comparison of system architecture and data sources

This section aims to provide a summary comparison of the key elements of the underlying system architectures used in the three countries as well as providing information on data sources used to populate these systems.

**Fig. 5:**
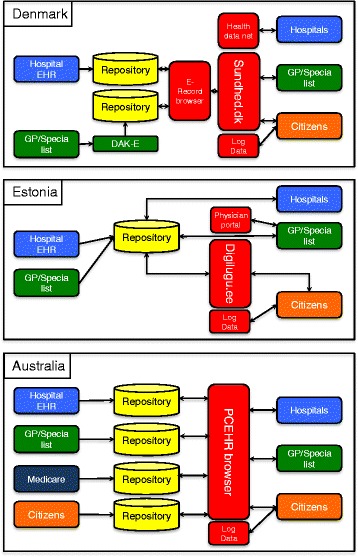
System architecture for citizens’ access to health data in Denmark, Estonia and Australia. The data input/creation is shown to the left, and the data output/viewing is shown to the right. (Figure made by the authors

In presenting the diagrams in Fig. 5 every effort has been made to map similar architectural components across the three countries using a common colour schema to represent data repositories, citizens, health professionals, other databases/systems and the citizen data portal:

##### Denmark

The top drawing in Fig. 5 shows the basic architecture of the Danish citizens’ access to health data. Data is created at the GP or specialist’s office, in the hospital patient administrative systems (PAS), and the hospital EHR systems. Data from the majority of the GPs are submitted to the Danish Quality Unit of General Practice (DAK-E), who makes it available to patients via Sundhed.dk. Data from hospital PAS and EHR systems are transferred to a repository database – the E-Record database and made available to citizens, GPs and other hospitals. Patient data are delayed two weeks for ethical reasons, and health care providers are only allowed to access data on the patients they treat. The citizens and the GPs get access to data via the Sundhed.dk and the hospitals access the E-Record browser via the secure National Health Data Network.

In Denmark, all hospitals are by law obliged to send care summaries to the central repository and this data is also visible in the patient portal. All medicine prescriptions except drugs prescribed during hospital admissions are all available through the Patient Portal.

##### Estonia

The center drawing in Fig. 5 illustrates the architecture of the Estonian Health Information System. Data is created either in hospitals or GP/specialist information systems and stored in the national EHR. Prescription data is stored in the Prescription Centre. Additional information about the citizen is collected from other state registries and accessible to both the health care providers and citizens through the PP, for example contact details and existing health insurance.

The data collected from different sources is presented to citizens, GPs, hospitals and clinics through the patient portal called *digilugu.ee*. However, most hospital information systems and some GP information systems have integrated the PP so that physicians do not need to access the web-based solution. The information is presented without a delay. In addition, specialists unable to afford their own information system can access e-health data through a government-provided Physician Portal.

All document transfer uses HL7 v3 (extended) standard, medical documents are kept in the XML format (HL7 CDA). Moreover, all structured data fields have object identifiers (OIDs). The X-road infrastructure with the smart ID-card provided authentication is the backbone of e-health services in Estonia used by both citizens and health care providers alike.

In Estonia, all health care providers are by law obliged to send care summaries to the central repository and this data is also visible in the patient portal. Digital prescriptions are all available in the Patient Portal.

##### Australia

The bottom part of Fig. 5 shows the basic architecture of the Australian MyHR. Clinical data are generated and held in GP, specialist, and hospital systems, with summary data then transferred to the MyHR system. A range of different data sources, both public and private, is used to provide a comprehensive summary record. The central MyHR infrastructure manages the location and transfer of data from the distributed system. There are a number of repositories that have been established to collect and store that clinical data but also there are links to other organizational repositories but there is no ability to directly query individual organizational EHRs, only the summary data exchanged with the repositories is provided through MyHR [10].

In Australia obligations to submit data to the central repository is currently limited but pressure from consumers is increasing.

#### Summary comparison of security, privacy and user support approaches for citizen access

##### Security

Every Danish citizen has since 1968 been given a unique personal identifier at birth (CPR-number). It consists of the 6 digit birthdate and a 4 digit check number where the last digit indicates the gender – uneven for male and even for female. After entering the CPR number as username and a self-chosen password the user is asked to enter an additional six-digit security number, called NEM-ID. Citizens can obtain a NEM-ID by personal appearance in a Bank, the municipal administration, or on-line by submitting their passport number for identification. The NEM-ID code is read from a (paper or electronic) token. This two-factor authentication is used for log-in to a number of public e- services and net-banking. Health professionals also have access to the personal health data via the portal Sundhed.dk, but are only allowed to access it when they are treating the person.

In Estonian public and private e-services are based on a common data transport middleware called the X-road. It is an organizational and technical environment that enables to connect multiple data sources and securely exchange data between institutions and people [13]. All citizens and health care providers can access the Patient Portal using the Estonian public key infrastructure (PKI). It provides every citizen with an encrypting key pair. The first, public is for encryption and the private one is for decryption. The PKI is in turn connected to an electronic identity, i.e. ID card or mobile ID. Every Estonian can have an ID-card from the first day they are born. ID cards are used for both regular and electronic identification as well as digital signatures. The ID card is the electronic key to using all Estonian public and private e-services based on the X-road [13].

In Australia, accessing the portal is only possible for those that have opted-in to the service. A person, above the age of 14, is eligible to be registered if they have been assigned a healthcare identifier and they have provided suitable proof of their identity to the System Operator. People can apply to register for an eHealth record via: telephone; face-to-face at Department of Human Services service centres; mail; online (www.ehealth.gov.au); and assisted registration by healthcare provider organisations. For minors under the age of 18, a parent or guardian can apply to register by providing evidence of their parental responsibility. Additionally, a ‘person responsible’ can apply to register an adult who is deemed not capable of making their own decisions by providing evidence of their authority to act on behalf of that person. Health care providers and their organisations are also required to register to access MyHR. Once registered a unique identifier is assigned to each user.

##### Privacy

In all three countries all activity is logged and the log files are accessible to the citizen. The patients can to monitor their log file and report any irregularity. In Denmark, a letter is sent to the citizen if a GP or specialist without any treatment relation has accessed the citizen. Treatment relations are detected as 1) hospital admission (PAS system), 2) general practitioner (reimbursement data), or 3) specialist visited after referral (record in national referral database). To supplement this, a bi-annual audit is performed in Denmark where a random sample of log files is checked for irregularities. A low number of cases of abuse are detected every year, and a fraction of these are so serious they lead to a sentence. In Denmark citizens have the right to block any information from access by health professionals - e.g. specific drugs prescribed or specific diagnosis.

In Estonia patients are also able to opt out of having their information in the Patient Portal. It is possible to close individual documents, for example information about a visit to a certain health professional or a diagnosis. Alternatively, the patient can close the entire medical record. The only data that the patient is not able to close is the so-called time-critical data - this includes allergies, medical procedures over the past month, last visit to the doctor or hospitalisation.

In Australia, the MyHR system is opt-in so patients, health care providers and health care provider organisations all need to register for their information to be included in the MyHR. Patients can manage access to their records through the patient portal, that is grant or deny others access. The documents and information stored on My Health Record are completely under consumer control. Consumers have the ability to hide clinical or Medicare documents and restore hidden documents. If documents are hidden from My Health Record, by a consumer, this information will not be accessible, even in an emergency. Audit of all access is logged in the system and this is available to the patient through the patient portal, but only at an organisational level so does not show which individual clinician has accessed the record. Patients can also set up notifications via SMS or email when certain activities occur.

For all countries, it is only possible to close the access to data, which can be reopened. It is not possible to erase data.

##### User support

In Denmark, the public health portal “Sundhed.dk” provides help desk to users with questions to technical and navigation problems. Questions regarding health issues are directed to the health professional or institution that entered the data. Similarly, the E-Health Foundation in Estonia has a user support phone and e-mail address available for citizens. However, no online helpdesk service is currently provided. The E-Health Foundation website has additional materials available for people to educate themselves on safe online behaviour and security matters. These include recommendations about not leaving the computer unattended when logged in to the PP or not leaving one’s ID-card unattended.

In Australia, a help desk facility is available for users who are experiencing technical difficulties. There is a range of learning modules, help functions and links to frequently asked questions and further information available within MyHR and on the website www.ehealth.gov.au *.*

#### Summary comparison of citizen adoption and use

This section presents and compares adoption and use by citizens of the e-portals. Every effort has been made to provide the most up-to-date data available in the three countries for the period January to December 2015.

Data could be collected and compared about:

1. Number of citizens who can have access (E.g. in Denmark it would be those who have a NEM-ID code, in Australia those who have opted-in)
2. Their age & gender.

**Fig. 6:**
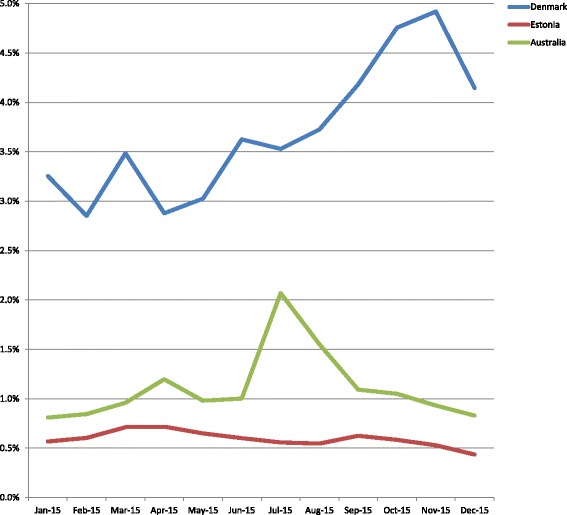
Number of citizens who have logged-in relative to number of citizens who can log in (DK: *n* = 4.154.733, EST: *n* = 1.313.271, AUS: *n* = 2.533.378)

Figure 6 provides a summary comparison of login data by citizens in the three countries during 2015.

**Fig. 7:**
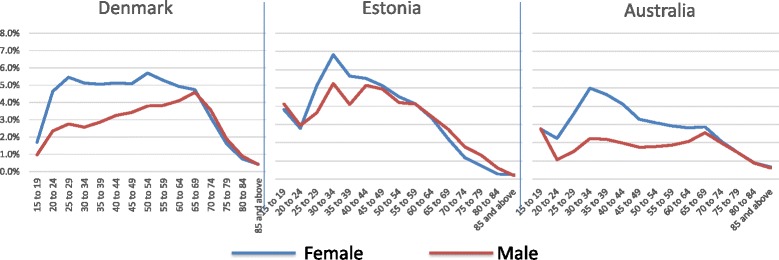
Age and gender distribution of citizens who logged-in [17] [18] [19])

Figure 7 presents a summary comparison of log-in data sorted by gender in different age groups across the three countries.

## Discussion

Based on the data and summary comparisons that have been presented above, it is evident that there are numerous similarities and differences in the type and level of access citizens have to their own health data in the three countries. In exploring these issues the discussion adopts a citizen-centred perspective to consider how the different e-portal systems support, protect and structure citizen interactions with their own health data, As a result this discussion is structured in three sections: Security, privacy and data protection; User Support; Citizen adoption and use.

Before commencing the discussion, it is important to note that there has been no attempt in this research paper to compare costs in relation to the development, implementation and/or on-going delivery of these different e-portal systems. Preliminary examination of the available data on costs across the three countries highlighted that even comparisons of ‘total costs’ were likely to be misleading and unhelpful in terms of contributing to the foci of the other analyses being presented.

### Security, privacy and data protection:

The analysis above highlights a key difference in citizen access relates to the opt-out approaches of Denmark and Estonia versus the required opt-in for the Australian system. While citizens in Denmark and Estonia can have a degree of confidence that the security, privacy and management of their data is automatically aligned to existing National and European regulations, the Australian opt-in presumes citizens understand and consent to the specific conditions of the system’s use.

This difference may not currently appear very significant. However, it is interesting to consider how the evolving landscape of ‘medical devices’ including those available through smart phone software applications is changing how data on a citizen’s health is being collected and analysed in real-time. Just as many citizens have become increasingly concerned by user agreements embedded in many social media platforms, identifying where the burden of responsibility lies for understanding what has been opted-in to will continue to shift in complex ways. Interestingly it is within the EU, and not Australia, where the most advanced discussions on both the impact of mobile health and emerging medical devices and consumer health applications is advancing most rapidly (HTTP://ec.europa.eu).

It can also be questioned whether the patient data can be copied or pasted into the providers own IT system, and for all the data that has been generated by health professionals it is as with any health record - paper or digital - an inter or intra professional communication is stored in the health providers system. The patient generated data will not automatically be stored in provider systems.

### User support

Differences in the way citizens are supported in the use of the e-portals across the three countries were identified. These included differences in terms of the communication methods available (i.e. whether assistance is available on-line, via email and/or phone help-line, and in the nature and content of the support services available from materials covering technical issues, through FAQs to resources on safe on-line behaviour.

The primarily technical focus of most of the user support is interesting when reflecting on the primary goal of providing citizens with access to their own health data. While in Denmark, questions regarding health issues are directed to health professionals, there appears not to be a highly structured approach in any of the three countries to grappling with issues arising from variations in citizen health literacy. These issues appear not to have been formally addressed in how support services have been developed and structured (i.e. the presumption of a degree of technical literacy appears to be implicit) and or how they will be understood and interpreted by different types of citizen users. Across the three countries there appears also to be limited user support for opt-in or opt out activities and limited support to ensure on-going user engagement with the e-portal services.

In this regard, it is useful to note that it was not possible in any of the three countries to obtain accurate user experience data. Indeed, it is unclear whether in any of the countries this type of data is being collated and analysed to improve user support services. These points neatly align with discussion in the final section of the discussion on citizen adoption and use of these e-portal systems.

### Citizen adoption and use

The data presented on citizen adoption and use clearly highlights significant differences in total users arising as a result of opt-in versus opt-out enrolment between Denmark and Estonia on one side and Australia on the other. More interestingly however, this analysis also shows that when examining log in data differences across gender and actual use are not dramatically different, even though Denmark appears to be leading the way.

Ultimately what can be learned from this adoption and use data? What are the legitimate conclusions that can be drawn from the number of times citizens log in and the number of unique visits they make correlated with variables such as age and gender?

Returning to this paper’s conceptual framework, it would appear that analyses of data both in each country and comparatively provide insights into both structure and process within Donabedian’s framework. The analyses however provide only limited insight into outcomes at both individual and population health levels. Given the complexity of the variables involved this is perhaps not surprising. However, it does highlight the need for more qualitative investigation of how investments in e-Health systems and e-portals providing citizen access to personal health data are actually contributing to patient empowerment and thereby improving individual and population health outcomes [14]. In this regard, two areas that are already being explored are health outcomes alongside MyHR use amongst rural and remote patients with chronic disease in Australia [11] and the development of citizen surveys for Danish users of their e-portal [15].

A potential source of bias to use log data to describe activities that will reflect in outcomes measures is that a log on to the portal might be to hide personal data or opt out of specific functions. However, this bias is estimated low as there is a continuous rise in the traffic on the pages.

## Conclusions

This research paper presents data on the type and level of access citizens have to their own health data in three countries. Following individual country presentations of data, the experiences of Denmark, Estonia and Australia were examined and similarities and differences explored. The discussion adopted a citizen-centred perspective to consider how the different e-portal systems support, protect and structure citizen interactions with their own health data in three key areas: Security, privacy and data protection; User support; and, citizen adoption and use.

The paper has highlighted the impact of opt-in/opt-out approaches on citizen access and the lack of a structured approach to addressing differences in citizen health and e-health literacy when using these e-portals. The research has also confirmed that while current data provides detail on the availability and use of personal health data by citizens, questions still remain over the ultimate impact on patient outcomes of these initiatives.

It is to be hoped that by presenting insights generated from the experiences of three countries at the fore-front of providing citizens access to their own health data this will prove useful both to improve initiatives in these three countries and guide other countries aspiring to support similar activities.

## Abbreviations

AUS: Australia
DK: Denmark
EST: Estonia
FTE: Full Time Equivalent
GP: General Practitioner
HIS: Health Information System
HMO: Health Maintenance Organization
ID: Identifier
MyHR: My Health Record
NHS: National Health Service
OID: Object Identifier
PAS: Patient Administrative System
PCEHR: Personally Controlled Electronic Health Record
PHR: Personal Health Record
PKI: Public Key Infrastructure
PP: Patient Portal
UK: United Kingdom
VA: Veterans Administration

## References

[1] PSFK. Google Health Fails To Bring Meaning To Data 2011. http://www.psfk.com/2011/06/frog-google-health-fails-to-bring-meaning-to-data.html (accessed 27 June 2017).

[2] Greenhalgh T, Stramer K, Bratan T, Byrne E, Russell J, Potts H. Adoption and non-adoption of a shared electronic summary record in England: a mixed-method case study. Br Med J. n.d.;2:647–52. doi:10.1136/bmj.c3111.. 10.1136/bmj.c3111 20554687

[3] NHS. UK, Health Space, Connecting for health. 2013. http://www.connectingforhealth.nhs.uk/systemsandservices/healthspace (accessed 27 June 2017).

[4] Mold F, de Lusignan S, Sheikh A, Majeed A, Wyatt JC, Quinn T (2015). Patients’ online access to their electronic health records and linked online services: a systematic review in primary care. Br J Gen Pract.

[5] Donabedian A. Evaluating the quality of medical care. Milbank Mem Fund Q. 1966; 5338568

[6] Sundhed.dk. Background 2016. https://www.sundhed.dk/borger/service/om-sundheddk/ehealth-in-denmark/background/ (accessed 27 June 2017).

[7] Kuivjogi K, Parre J, Tammaru T VT. Tervise Infosüsteemi Arengukava 2005–2008 (The Health Information system Development Plan). Tallinn: Estonian Ministry of Social Affairs; 2004.

[8] Lusignan S de, Morris L, Hassey A, Rafi I. Giving patients online access to their records: opportunities, challenges, and scope for service transformation. Br J Gen Pract 2013;63:286–287. doi:10.3399/bjgp13X668032. 10.3399/bjgp13X668032 PMC3662423 23735378

[9] Cummings E, Cheek C. The cradle coast personally controlled electronic health record evaluation research plan. Stud Health Technol Inform. 2012;178 22797013

[10] Pearce C, Bainbridge M (2014). A personally controlled electronic health record for Australia. J Am Med Informatics Assoc.

[11] Almond H, Cummings E, Turner P (2013). Australia’s personally controlled electronic health record and primary healthcare: generating a framework for implementation and evaluation. Stud Health Technol Inform.

[12] Bennett C (2009). A healthier future for all Australians: an overview of the final report of the National Health and hospitals reform commission. Med J Aust.

[13] Republic of Estonia ISA. X-ROAD Fact Sheet 2014. https://www.ria.ee/public/x_tee/X-road-factsheet-2014.pdf (accessed 27 June 2017).

[14] Showell C, Turner P (2013). The PLU problem: are we designing personal ehealth for people like us?. Stud Health Technol Inform.

[15] Bertelsen P, Tornbjerg K (2015). Danish citizens’ expectations to the use of eHealth. Stud Health Technol Inform.

[16] Ministry of Health. Healthcare in Denmark 2017. http://www.sum.dk/English.aspx (Accessed 27 June 2017).

[17] Danish health portal 2016. www.sundhed.dk (accessed 27 June 2017).

[18] Estonian health portal 2016. www.e-tervis.ee/index.php/en/ (accessed 27 June 2017).

[19] Australian health portal 2016. www.digitalhealth.gov.au (accessed 27 June 2017).

